# Alcohol and premature death in Estonian men: a study of forensic autopsies using novel biomarkers and proxy informants

**DOI:** 10.1186/1471-2458-12-146

**Published:** 2012-02-27

**Authors:** Inge Ringmets, Jana Tuusov, Katrin Lang, Marika Väli, Kersti Pärna, Mailis Tõnisson, Anders Helander, Martin McKee, David A Leon

**Affiliations:** 1 Department of Public Health, University of Tartu, Ravila 19, 50411 Tartu, Estonia; 2 Institute of Pathological Anatomy and Forensic Medicine, University of Tartu, Ravila 19, 50411 Tartu, Estonia; 3 Estonian Forensic Science Institute, Tallinn, Estonia; 4 Alcohol Laboratory, Department of Laboratory Medicine, Karolinska Institutet, C1:74, Clinical Chemistry, Karolinska University Laboratory Huddinge, S-141 86 Stockholm, Sweden; 5 ECOHOST, London School of Hygiene & Tropical Medicine, University of London, 15-17 Tavistock Place, London WC1H 9SH, UK; 6 Department of Non-communicable Diseases Epidemiology, London School of Hygiene & Tropical Medicine, University of London, Keppel St, London WC1E 7HT, UK

## Abstract

**Background:**

Alcohol makes an important contribution to premature mortality in many countries in Eastern Europe, including Estonia. However, the full extent of its impact, and the mechanisms underlying it, are challenging issues to research. We describe the design and initial findings of a study aimed at investigating the association of alcohol with mortality in a large series of forensic autopsies of working-age men in Estonia.

**Methods:**

1299 male deaths aged 25-54 years were subject to forensic autopsy in 2008-2009. The routine autopsy protocol was augmented by a more systematic inspection of organs, drug testing, assay of liver enzymes and novel biomarkers of alcohol consumption (EtG, EtS and PEth), together with proxy interviews with next of kin for deaths among men who lived in or close to a major town.

**Results:**

595 augmented autopsies were performed. Of these, 66% were from external causes (26% suicide, 25% poisoning). 17% were attributed to circulatory system diseases and 7% to alcoholic liver disease. Blood alcohol concentrations (BAC) of ≥ 0.2 mg/g were found for 55% of deaths. Interviews were conducted with proxy informants for 61% of the subjects who had resided in towns. Of these, 28% were reported in the previous year to have been daily or almost daily drinkers and 10% had drunk non-beverage alcohols. Blood ethanol and the liver enzyme GGT were only associated with daily drinking. However, the novel biomarkers showed a more graded response with recent consumption. In contrast, the liver enzymes AST and ALT were largely uninformative because of post-mortem changes. The presence of extremely high PEth concentrations in some samples also suggested post-mortem formation.

**Conclusion:**

We have shown the feasibility of deploying an extended research protocol within the setting of routine forensic autopsies that offer scope to deepen our understanding of the alcohol-related burden of premature mortality. The most unique feature of the study is the information on a wide range of informative alcohol biomarkers, several of which have not been used previously in this sort of post-mortem research study. We have demonstrated, for the first time, the epidemiological value and validity of these novel alcohol biomarkers in post-mortem samples.

## Background

Estonia is the most northerly of the three Baltic States, with a high burden of premature mortality, especially among men. In 2008, male life expectancy was only 68.7 years compared to the European Union average of 76.3 [1]. This gap is due, largely, to excess deaths among men of working age. Heavy alcohol consumption has been implicated as a major cause, with high levels of hazardous drinking in the population [2]. The amount of pure alcohol consumed per person in 2008 was 12.1 l [3]. Between 1994 and 2006, the proportion of men aged 25-64 years drinking more than 280 g equivalent of pure alcohol per week increased from 9.1% to 16.2% [4]. Non-beverage ethanol containing substances not intended for consumption (such as eau de colognes and medicines), that have been implicated in the high burden of premature death in neighbouring Russia [5,6], are also consumed in Estonia [7] and are widely available in parts of the capital, Tallinn [8].

Mortality from a range of conditions such as cirrhosis of the liver are known to be strongly linked in a dose-response fashion to alcohol intake [9].

The impact of drinking on mortality from cardiovascular disease does, however, remain controversial, particularly whether heavy and/or episodic drinking is a risk factor for deaths from ischaemic heart disease and, if so, what the mechanisms may be [10,11]. A recent study in Russia suggested that heavy consumption of spirits may be associated with fatal non-atherosclerotic damage to the heart (including frank cardiomyopathy), misclassified as ischaemic heart disease [12]. Further investigation of this hypothesis is a priority given the notably high levels of cardiovascular mortality in Estonia. In 2008, the age standardised death rate (age 0-64) from diseases of the circulatory system in Estonia was 195 per 100,000 (male) and 49 per 100,000 (female), compared with 76 and 19, respectively, in neighbouring Finland and 45 and 13, respectively, in Spain, one of the European countries with the lowest rates [1]. Similar levels of excess mortality are seen in other former-Soviet countries, which also exhibit particularly harmful patterns of alcohol consumption [2].

Researching the scale and nature of alcohol-related harm to health in populations characterised by episodic heavy drinking is challenging [13]. The classic cohort study, in which baseline alcohol drinking is related to deaths ascertained over an extended follow-up period of years or even decades, is not well suited to detecting acute alcohol-related deaths such as sudden cardiac death that may be precipitated by an episode of heavy drinking. While some alcohol-related deaths may be the end result of the cumulative effects of a long period of hazardous drinking, for others it may be the result of a relatively short period of very intense drinking. Many epidemiological studies of this topic also suffer from selection bias in recruitment and follow-up [14]. In addition, the heaviest drinkers are often socially marginalised and comprise a hard to reach population who are difficult to recruit and follow-up [15]. Finally, the selection of underlying cause of death when alcohol may be involved is a difficult issue, being potentially biased by cultural factors as well as having an arbitrary aspect due to the frequent presence of multiple alcohol-induced pathologies. To this extent, cause of death from the death certificate provides a very partial basis for ascertaining the full extent of the impact of alcohol on potentially fatal pathologies.

Given these difficulties triangulating data from several different sources and study designs may be the optimal strategy. While each has its problems and limitations, used in combination, they may provide important insights. A study of deaths subject to forensic autopsy provides one such source that has not been adequately exploited in Estonia or neighbouring countries. In this paper, we describe the design and first results of a research study of working age men subject to forensic autopsy in Estonia 2008-2009, with special emphasis on the behaviour of novel alcohol biomarkers and their relationship to proxy reports of drinking behaviour among the deceased.

## Methods/Design

The Estonian Forensic Study of Alcohol and Premature Death was based on deaths subject to autopsy by the Estonian forensic service. Its aims were to:

1 assess the prevalence of pathological and biomarker evidence of alcohol-induced damage and high levels of alcohol consumption in the days immediately prior to death;

2 investigate how far alcohol biomarkers and specific pathologies present at death are related to proxy reports of hazardous drinking in the deceased;

3 ascertain the potential contribution of alcohol consumption to death from a range of causes including sudden cardiac death and other cardiovascular disease.

The substantive results reported in this paper focus on the first two aims, i.e. the levels of novel alcohol biomarkers and the relationship between such biomarkers and reported drinking behaviours.

### Forensic autopsy in Estonia

Today, the forensic science service in Estonia is equipped to standards comparable with the rest of Western Europe, having undergone extensive modernisation since independence in 1991 following the collapse of the Soviet Union. Forensic autopsies are carried out in one of four Regional Forensic Departments belonging to the Estonian Forensic Science Institute. In Estonia a forensic medical autopsy is conducted in one of four circumstances: if there is evidence of a crime; where death appears to be caused by external factors (injury, poisoning or violence) but no crime is suspected; where the state of the body makes it impossible to assign a cause from external inspection; or where the identity of the deceased is unknown [16]. While such deaths comprise only a small (14%) and selective fraction of all deaths in Estonia in 2008-2009, they include a substantial proportion of those dying from acute effects of alcohol consumption (including all deaths attributable to alcohol poisoning, injuries and violence), as well as those from the marginalised social groups who are often underrepresented in other study designs.

### Study protocol

The protocol was developed drawing on our extensive experience as either forensic specialists or researchers investigating the link between alcohol and mortality in Russia. In the design stage, we undertook a review of the literature and also had extensive discussions with other colleagues conducting routine forensic autopsies in Estonia. The protocol went beyond usual forensic practice in a number of respects:

1. It was designed to record in more detail than usual the presence or absence of pathologies, with particular emphasis upon minor pathologies indicative of alcohol-related damage but may not have been directed implicated as a cause of death.

2. In addition to the routine measurement of blood and urine ethanol concentrations we assayed levels of liver enzymes, checked for the presence of a range of drugs regardless of cause of death, and analysed concentrations of alcohol biomarkers in blood and urine;

3. For men who lived in or near main cities, we endeavoured to interview relatives or friends of the deceased man to obtain information about their circumstances and drinking behaviour.

### Pilot study

In September 2007, a pilot study to assess the feasibility of using the enhanced protocol was undertaken in Tartu based on 30 forensic autopsies of men aged 40-54 years. Simultaneously questionnaire was developed that could be used to obtain information about the deceased from proxy informants. This instrument was adapted from earlier work in Russia, where this approach had been tested extensively, including comparisons with externally verifiable information from other sources [13]. The questionnaire was piloted on 10 proxy informants. Following these pilot studies, revisions were made to the protocol and study instruments before they were employed in the main study. The final version of the questionnaire was developed in Estonian together with a Russian version, as respondents were interviewed in their mother tongue which could be either language.

### Main study protocol

#### Target population

The study target population was all deaths in Estonia subject to forensic autopsy of men aged 25-54 years. For logistical reasons, the interview study was restricted to the subset of men who lived in or near one of the country's five main cities: Tallinn, Tartu, Pärnu, Kohtla-Järve and Narva.

#### Autopsy process

The enhanced protocol was implemented within the context of the routine work of the forensic service. Information sheets that set out inclusion criteria and information about taking and managing samples were posted on the walls of mortuaries in the four Regional Forensic Departments. Pre-prepared collection packs were assembled and distributed to each department. Each pack contained a copy of the enhanced autopsy protocol, data collection proforma and sample containers, each labelled with a unique bar code. The samples from outlying departments were dispatched to Tartu after the autopsy (see below) where they were processed. Completed autopsy protocols from which the name of the deceased was removed were sent to Tartu and checked by one of the authors (MV), a senior forensic pathologist.

#### Information collected at autopsy

The autopsy protocol was completed retrospectively by the forensic specialist who had undertaken the autopsy. A summary of the types of information collected is shown in Table 1.

**Table 1:** Information collected at autopsy

General	
Identifiers	study number, forensic autopsy protocol number;
Time	date and time of death, date and time of autopsy, date and time blood sample taken;

**Macroscopic examination of organs**	

Heart	weight, length, breadth, depth, thickness of left and right ventricular walls, thickness of intraventricular septum; evidence of acute infarction and/or old infarction, other gross cardiac anomaly, atheromatous occlusion of coronary arteries, appearance of coronary arteries, thrombus of coronary arteries, other cardiovascular system;
Kidneys	renal disease;
Lungs	pneumonia, aspiration of gastric contents, active tuberculosis, healed tuberculosis with scarring, other (specify);
Liver	weight, length, right and left lobe height and depth; fatty liver (focal and diffuse), fibrosis, cirrhosis, other (specify);
Pancreas	acute and chronic pancreatitis, other (specify);
Oesophagus, stomach and duodenum	gastritis, gastric ulcer, duodenitis, duodenal ulcer, oesophageal varicosities, oesophagitis, other (specify);
Brain	weight; haemorrhage, infarction, new contusions, old brain trauma, cortical atrophy, cerebellar atrophy, other (specify);

**Laboratory investigations**	

Ethanol concentration	alcohol concentration in blood, vitreous humour and urine
Ethanol biomarkers (subset only)	Phosphatidylethanol (PEth) in blood, ethyl glucronide (EtG) and ethyl sulphate (EtS) in urine
Other alcohols and ethanol substitutes	methanol, isopropanol, isobutanol, isoamyl alcohol, ethylene glycol in blood and urine;
Drugs	morphine, fentanyl, marijuana, amphetamine, cocaine, buprenorphine, methadone, other (specify);
Liver enzymes	gamma-glutamyl transferase (GGT), alanine and aspartine aminotransferase (ALT and AST);

**Histology**	

Heart	right and left ventriculum and atrium, septum intraventricularis;
Coronary artery	tissue sample taken in case of thrombosis;
Liver	right and left lobe;
Pancreas	parenchyma, any lesion;
Brain	cortex, cerebellum, basal ganglia, mamiliary bodies;
Kidneys	right, left;
Lung	parenchyma, any lesion;
Stomach and duodenum	lesion;
**Cause of death**	immediate, intermediate, underlying, external, other important diseases, all coded to ICD-10; manner of death.

#### Cause of death

The underlying cause of death, and up to three associated causes of death as determined by the forensic pathologist was recorded and coded according to the 10^th ^revision of the International Classification of Diseases (ICD-10). At the point of determining cause of death, in most cases the only information regarding alcohol consumption of the deceased was based on evidence of alcohol-induced pathology found at autopsy, and alcohol concentrations of blood and urine. These latter laboratory results are sometimes only available after the cause of death was first determined. If these results were contrary to what was initially assumed, this would then lead to revision of the cause of death. An example of this might be where a diagnosis of sudden cardiac death might be revised to acute alcohol poisoning if the blood or urine alcohol concentrations came back above the threshold considered as lethal. The detailed results of the novel biomarker assays and proxy interviews collected for this study were not available to the forensic pathologist.

#### Biological samples

Blood was collected from peripheral vessels, usually the femoral vein, using a syringe and injected into vacutainer tubes (two 10-mL EDTA and one 10-mL plain). Urine was taken directly from the bladder with a syringe. Vitreous humor was aspirated from both eyes using a syringe. A sample of renal tissue (~50 g) was taken to provide a source of DNA for future studies. Samples were initially refrigerated at 4°C before being transferred to the forensic laboratory in Tartu for processing and analysis. Samples from locations outside Tartu were transported there in ice-packed cold boxes, normally arriving within 15 h.

In Tartu, one EDTA and one plain tube were centrifuged (2100 rpm) for 15 min at 4°C and the plasma and serum collected. According to the amount of material available up to eight bar-coded cryotubes were filled for each case: two each with whole blood, plasma, serum and urine. One cryotube of each specimen was stored at -80°C to provide a resource for further analysis. The renal tissue samples were stored at -20°C.

Urine and whole blood samples were sent on dry ice to the Karolinska Institutet, Stockholm for analysis of novel alcohol biomarkers that in live subjects have been shown to reliably detect moderate or heavy alcohol consumption up to several weeks previously. However, as these assays were labour intensive and time consuming, only a subset of samples were sent. As we wished to see what these biomarkers added to post-mortem measures of ethanol, we sent the 245 blood samples and 142 urines for subjects where blood or urine ethanol concentrations had been determined and a vitreous humour sample obtained.

#### Laboratory assays

Urine and blood alcohol concentrations (BAC) were measured in Tartu by headspace gas chromatography. To detect evidence of liver damage, serum aspartate aminotransferase (AST), alanine aminotransferase (ALT) and gamma-glutamyl transferase (GGT) activities were also measured. ALT and AST were measured using the kinetic photometric method and GGT with the kinetic colorimetric method in a Hitachi 2000. The reference values (for live persons) are as follows: ALT < 41 U/L, AST < 38 U/L and GGT < 61 U/L [17].

Several alcohol biomarkers were measured at the Karolinska Institutet. Whole blood phosphatidylethanol (PEth) is a test for moderate to heavy alcohol consumption over the past ~1-3 weeks and is determined by combined liquid chromatography and mass spectrometry (LC-MS) [18]. Urine ethyl glycuronide (EtG) and ethyl sulphate (EtS) [19] are conjugated minor ethanol metabolites used to indicate consumption of alcohol in the past ~1-2 days, also determined by LC-MS [20]. These biomarkers have the added advantage of not being affected by the extensive genetic variation in the main ethanol metabolism pathways. The limits of quantitation (LLOQ) used to indicate a positive test result were 0.1 μmol/L for total PEth, 0.5 mg/L for EtG and 0.1 mg/L for EtS. For total PEth, a cutoff at 0.7 μmol/L is used to distinguish moderate from excessive drinking [21]. It had also been intended to measure serum carbohydrate-deficient transferrin (CDT), a biomarker of heavy alcohol consumption over recent weeks, but this was not possible because of the high prevalence of haemolysis of the serum samples that would have interfered with the analyis [22].

Urine samples were screened in Tartu for narcotics, using a rapid urine test (Multiscreen 10 MTD; Biomedical Diagnostics). The LLOQ used to indicate a positive test result were 1000 ng/mL for amphetamine, methamphetamine and tricyclic antidepressants, 300 ng/mL for cocaine, opiates, methadone, barbiturates and benzodiazepines, 50 ng/mL for Δ- tetrahydrocannabinole, 500 ng/mL for ecstasy and 10 ng/mL for buprenorphine. Where screening results were positive, further confirmatory analysis was undertaken using combined gas chromatography and mass spectrometry (GC-MS).

#### Interview study

Within two weeks of the forensic autopsy protocols and samples arriving in Tartu, details of next-of-kin were sought from the forensic departments. After a minimum of two months following the death (an interval determined by cultural norms concerning the period of mourning) the deceased's next-of-kin were contacted. Following a brief explanation about the study, they were asked if they, or someone else would be willing to be interviewed about the deceased's life. In order to maximise the validity of the information provided, pre-defined criteria were used to select the best informant when several were available. If the next-of-kin was not the best proxy, or refused to be interviewed, contact was then made with another proxy. Those who had had daily contact with the deceased during his last year of life were preferred. In addition, a priority list was used to select the person likely to have the most intimate knowledge of the deceased's life. This was based on their formal relationship, which in declining order of preference was the widow or partner of the deceased, sister (over 18 years), mother, brother (over 18 years), father, child (over 18 years) or finally other. Once agreement and signed consent was obtained an interview with the selected proxy was undertaken by a trained researcher.

#### Information collected at interview

The interview questionnaire focussed on collecting information on the socio-demographic characteristics and health behaviours of the deceased, as summarised in Table 2. Particularly detailed information was collected about alcohol consumption, with a reference period of the year preceding the man's death.

**Table 2:** Information collected at interview

General information	Information recorded
Respondent	Age, date of birth, place of birth, nationality, marital status, number of children, education, profession, views about his/her area of residence;
The deceased and his family at time of death	Composition and structure of household, description of the home of the subject (number of rooms, amenities, properties), economic situation and income of the household, whether/when his parents had died;
Relationship of res- pondent to deceased	Relationship to the subject, duration and capacity of knowing the subject (special reference of knowing him in the last year before his death);
Subject	Age, date of birth, place of birth, nationality, marital status, number of children, education, profession, the main reason for ceasing regular paid employment (if applicable); History of serving in the army, serving in a zone of conflict, having been to prison;
	Relations in the family, having close friends, being involved in physical fights during the last year, diseases and disabilities (special reference in the last year before his death), persistent large changes in the subject's circumstances and/or behaviour (diet, exercise, drinking, smoking) that have occurred because of ill health or disability;
	Duration and pattern of smoking if applicable;

**Questions regarding drinking of alcohol by the subject**	**Information recorded**

Frequency and amount of drinking	Frequency and days of week of drinking beer, wine, spirits, surrogates, homemade samogon, alcoholic coctails; Usual and maximum amount of beer, wine, spirits, surrogates, homemade samogon, alcoholic coctails drunk on one occasion (special reference to drinking during the last year before his death);
Indication of hazardous drinking	Drinking spirits together with either beer or wine at the same sitting, drinking large quantities of spirits without also eating some food at the same sitting, frequency of becoming excessively drunk/having hangover, drinking alcohol before noon, frequency of failing to fulfil his family or personal or work obligations due to drinking alcohol, going to sleep at night with his clothes on because of being drunk, drinking alone, frequency and duration of *zapoi*, being arrested because he was drunk, drinking surrogates;
Other alcohol questions	Distance of get to the nearest place where one can buy beverages, being admitted to hospital/clinic because of alcohol poisoning, having had help or advice from a doctor, narcologist, social worker or some other professional for an alcohol problem, subject's father when the subject was growing up or anyone in the subject's household apart from him going on *zapoi*;
Surrogates	Main reason and time of start of drinking surrogates, drinking surrogates at home, types and amount of surrogates drunk, subject's father when the subject was growing up or anyone in the subject's household apart from him drinking surrogates;

#### Data entry and processing

Data from the autopsy protocols and questionnaires were double-entered and range and consistency checks conducted.

#### Statistical analysis

Descriptive statistics were mainly presented as frequency tables. Medians with interquartile ranges (IQR) or ranges were used for presenting the results of biomarkers. Fisher or chi-squared test was used for assessing the association between different characteristics (age, cause of death, BAC) and inclusion/exclusion to the study or whether a proxy interview was obtained. Comparing biomarker levels between different proxy-reported alcohol drinking frequencies, *t*-test or linear regression was used with log transformed biomarker values. The data were analysed using the statistical package Stata 10 [23].

#### Ethical approval and data protection

The study was approved by the Ethics Review Committee on Human Research at the University of Tartu, Estonia (No. 154/22, 20.11.2006) and the processes for handling personal data were registered with the Estonian Data Protection Inspection. The study was undertaken with the full support of the Estonian Forensic Science Institute that has sole statutory responsibility for forensic autopsies in Estonia.

## Results

**Figure 1:**
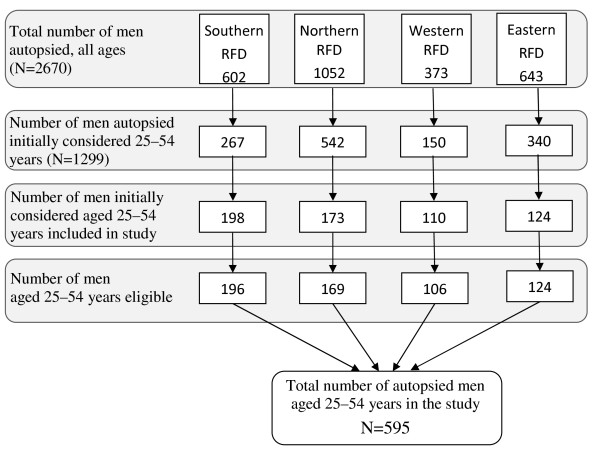
**Selection of study subjects by Regional Forensic Departments (RFD) of the Estonian Forensic Science Institute**. All four Regional Forensic Departments in Estonia participated in the study. During the study period (15 January 2008-11 September 2009), 3427 autopsies were performed by the Estonian Forensic Science Institute, 2670 (77.9%) of which were male. Of these, 1299 subjects were initially determined to be within the designated age range of 25-54 years.

All four Regional Forensic Departments in Estonia participated in the study. During the study period (15 January 2008-11 September 2009), 3427 autopsies were performed by the Estonian Forensic Science Institute, 2670 (77.9%) of which were male. Of these, 1299 subjects were initially determined to be within the designated age range of 25-54 years. The selection of subjects into the study is shown in Figure 1.

The aim was to include all consecutive eligible autopsies in the study. However, due to periodic high routine workload, only a subset of 605 of the total of 1299 eligible autopsies were included. Following initial inclusion, 8 deaths were found to be outside the designated age range and the identity of a further two could not be established. The final recruitment rate was thus 46% (595/1299).

The characteristics of the forensic autopsies included and excluded from the study are compared in Table 3. As expected, due to workload of the forensic specialist being the primary driver for exclusion, there were no significant differences with regard to age group. However, there were slightly more deaths from external causes and with high blood alcohol concentrations among those included.

**Table 3:** Characteristics of eligible deaths included and excluded from the study

Characteristics	Included		Excluded		
	
	Number	%	Number	%	*P *value*
**Age (years)**					0.416
25-34	144	24.2	186	26.4	
35-44	159	26.7	198	28.1	
45-54	292	49.1	320	45.5	
Underlying cause of death**					0.001
Diseases	184	30.9	205	29.1	
*Circulatory system (I00-I99)*	*101*	*54.9*	*103*	*50.2*	
*Digestive system (K00-K99)*	*54*	*29.3*	*63*	*30.7*	*0.591*
*Other diseases*	*29*	*15.8*	*39*	*19.0*	
Unknown causes (R95-R99)	16	2.7	50	7.1	
External causes (V01-Y98)	395	66.4	449	63.8	
**Blood alcohol concentration (BAC)**					0.013
No	31	5.2	63	8.9	
Yes	564	94.8	641	91.1	
*BAC < 0.2 mg/g*	*254*	*45.0*	*354*	*55.2*	*< 0.001*
*BAC ≥ 0.2 mg/g*	*310*	*55.0*	*287*	*44.8*	

**Total**	**595**	**100**	**704**	**100**	

* Comparing distribution of different characteristics between deaths included and excluded from the study using chi-squared test

** As coded by the forensic specialist following autopsy

### Cause of death

The distribution of included deaths by underlying cause of death is shown in Table 4. By far the largest single group of deaths by chapter were those attributed to external causes which accounted for two-thirds of the total. Of these, suicides and poisoning with alcohol or other substances each constituted a quarter. Deaths due to diseases of the circulatory system comprised nearly one in five of all deaths, of which ischaemic heart disease was the major single component. Alcoholic liver disease accounted for most of the deaths of from diseases of the digestive system. There was variation by cause in the proportion of deaths where BAC levels of ≥ 0.2 mg/g were detected: 34% circulatory diseases, 33% digestive diseases, 67% external causes, 11% other diseases and 63% of unknown causes, although the latter two percentages are based on particularly small numbers.

**Table 4:** Distribution of deaths included in the study by underlying cause of death*

Cause of death by major groups (ICD-10 code)	Number	%
**Diseases**	**184**	**30.9**
*Circulatory system (I00-I99)*	*101*	*54.9*
Ischaemic heart diseases (I20-I25)	42	41.6
Alcoholic cardiomyopathy (I42.6)	3	3.0
Cardiomyopathy (I42, excl I42.6)	6	5.9
Cerebrovascular diseases (I60-I69)	14	13.9
Other diseases of the circulatory system	36	35.6
*Digestive system (K00-K99)*	*54*	*29.3*
Alcoholic liver disease (K70)	44	81.5
Cirrhosis and fibrosis (K74)	1	1.9
Other diseases of the digestive system	9	16.7
*Other diseases*	*29*	*15.8*
**Unknown causes (R95-R99)**	**16**	**2.7**
**External causes (V01-Y98)**	**395**	**66.4**
Transport Accidents (V01-V99)	38	9.6
Falls (W00-W19)	23	5.8
Accidental drowning and submersion (W65-W74)	28	7.1
Inhalation of gastric contents or food causing obstruction	34	8.6
of respiratory tract (W78-W79)		
Alcohol poisoning (X45)	45	11.4
Accidental poisoning by and exposure to noxious	53	13.4
substances (X40-X49, excl X45)		
Intentional self-harm (X60-X84)	101	25.6
Assault (X85-Y09)	30	7.6
Exposure to excessive natural cold (X31)	10	2.5
Event of undetermined intent (Y10-Y34)	20	5.1
Other external causes	13	3.3

**Total**	**595**	**100**

* As coded by the forensic specialist following autopsy

### Biomarkers

The numbers of deaths for which each alcohol-related biomarker were measured are shown in Table 5. Of the 595 deaths included, blood samples were obtained for 564 (94.8%) and urine samples for 437 (73.4%). However, the number of usable serum samples obtained (365, 61.3%) was smaller than the total number of blood samples taken because the blood was haemolysed or putrefied or in some other way unusable. All tests gave results with very wide ranges, but the medians were considerably higher than the corresponding reference intervals and a large proportion tested positive for recent drinking.

**Table 5:** Alcohol biomarker availability and levels

Biomarker, units	Number	% of all autopsies (N = 595)	Median	Range
Ethanol (blood), mg/g	564	94.8	0.78	0-6.59
Ethanol (urine), mg/g	437	73.4	1.51	0-6.14
AST (serum), U/L	364	61.2	1014	59-28790
ALT (serum), U/L	364	61.2	792	8-42580
GGT (serum), U/L	365	61.3	92	15-1884
PEth (blood), μmol/L	245	42.2	9.8	0-121.5
EtG (urine), mg/L	157	26.4	79	0-2699
EtS (urine), mg/L	157	26.4	22	0-480

About 80% of the whole blood samples and urines examined tested positive for total PEth (> 0.7 mlL and EtG/EtS, respectively. It should be noted that some of the PEth values were considerably higher than those observed in studies of chronic heavy drinkers [18]. In fact 28% of the blood samples showed total PEth values of 22 μmol/L or above, with 4.5% greater than 50 μmol/L, suggesting post-mortem formation [18].

### Proxy informant interviews

**Figure 2:**
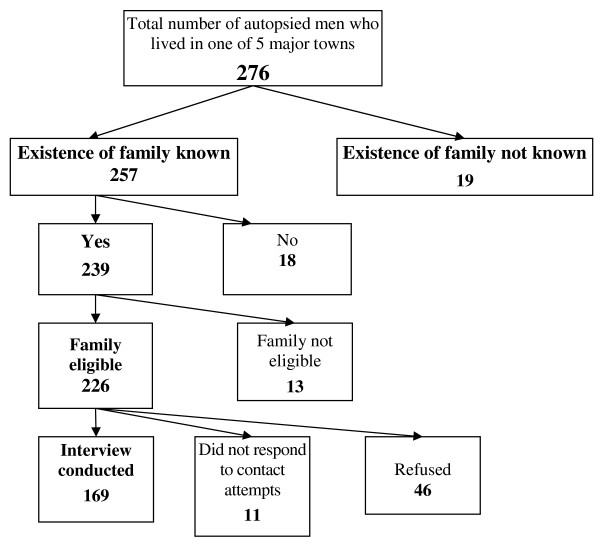
**Flow chart of subjects for proxy informant interview**. Of 276 deceased men eligible for inclusion because they lived in one of the 5 major towns in Estonia, for 7% it was not possible to find out whether they had had a family as their bodies were either collected by the funeral agency or their funeral was organised by the local authorities. Of those for whom it was possible to determine whether they had a family, 7% had no family (lived alone). Of the remaining 239 deceased men, 95% had families eligible for interview, i.e. at least one family member had had daily contact with the deceased during the last year before his death. Of these, a proxy interview was conducted for 75%, while 20% of families refused, and 5% did not respond to contact attempts. Overall we obtained proxy interviews for 61% (169/276) of all eligible deceased men, representing 75% of all where we knew that there were eligible proxy informants (169/226).

The process of selection of subjects for proxy informant interview is shown in Figure 2. Of 276 deceased men eligible for inclusion because they lived in one of the 5 major towns in Estonia, for 7% it was not possible to find out whether they had had a family as their bodies were either collected by the funeral agency or their funeral was organised by the local authorities. Of those for whom it was possible to determine whether they had a family, 7% had no family (lived alone). Of the remaining 239 deceased men, 226 had families eligible for interview, i.e. at least one family member had had daily contact with the deceased during the last year before his death. Of these, a proxy interview was conducted for 169 (75%), the remainder either refusing (20%) or did not respond (5%), resulting in a response rate of 61% (169/276). The distribution of the relationship of the proxy to the subject was wife/girlfriend/partner (n = 47) and parent (n = 46; 35 mothers and 11 fathers), sister (n = 16), other close relative (n = 12), other companion (n = 12), brother (n = 11), son (n = 11), daughter (n = 7), friend (n = 7).

The characteristics of those deaths where a proxy interview was obtained are compared in Table 6. Those for whom a proxy interview was obtained were less likely to have died from diseases of the digestive system. In addition, those deaths for whom a proxy interview was obtained were less likely to have a BAC ≥ 0.2 mg/g than those where a proxy was not interviewed.

**Table 6:** Characteristics of deaths in the study and eligible* to be interviewed according to whether a proxy interview was obtained

Characteristics	Included		Excluded		
	
	Number	%	Number	%	*P *value**
**Age (years)**					0.291
25-34	50	29.6	29	27.1	
35-44	35	20.7	31	29.0	
45-54	84	49.7	47	43.9	
**Underlying cause of death**					0.769
Diseases	70	41.4	40	37.4	
*Circulatory system (I00-I99)*	*41*	*58.6*	*21*	*52.5*	
*Digestive system (K00-K99)*	*10*	*14.3*	*16*	*40.0*	*0.002*
*Other diseases*	*19*	*27.1*	*3*	*7.5*	
Unknown causes (R95-R99)	4	2.4	2	1.9	
External causes (V01-Y98)	95	56.2	65	60.7	
**Blood alcohol concentration (BAC)**					0.788
**measured**					
No	8	4.7	5	4.7	
Yes	161	95.3	102	95.3	
*BAC < 0.2 mg/g*	*89*	*55.3*	*49*	*48.0*	*< 0.001*
*BAC ≥ 0.2 mg/g*	*72*	*44.7*	*53*	*52.0*	

**Total**	**169**	**100**	**107**	**100**	

*Only for deaths to men resident in and around one of the five major towns attempts were made to undertake proxy interview

**Comparing distribution of different characteristics between deaths included and excluded from the study using chi-squared or Fisher test

### Socio-demographic and behavioural characteristics of subjects

The characteristics of the deceased men, based on information provided by a proxy informant, are shown in Table 7. Over half the deceased men with proxy interviews were of non-Estonian nationality (mainly Russian), a half were separated, divorced or had never been married, while just under a half were in a regular paid job. The proxy reported prevalence of drinking at all in the past year was 84% and in the past month 54%, with over a quarter of men reported to have been drinking daily or almost daily in the year before death. One in 10 of the deceased were reported by proxies to have drunk non-beverage alcohols at least several times in the year before death.

**Table 7:** The characteristics of the deceased men based on information provided by a proxy informant

Characteristics	Number	%
**Age group (years)**		
25-34	50	29.6
35-44	35	20.7
45-54	84	49.7
**Nationality**		
Estonian	68	40.2
Russian	86	50.9
Other	15	8.9
**Marital status before death**		
Living with wife in registered marriage	45	26.6
Living with partner not in registered marriage	33	19.5
Divorced or living separately	42	24.9
Widowed	4	2.4
Never been married	45	26.6
**Employment status**		
In a regularly paid job	78	46.2
In a temporary job	26	15.4
Unemployed, looking for job	23	13.6
Unemployed, not looking for job	19	11.2
Unemployed and disability pension	12	7.1
Disability pension (incl 1 retired person)	11	6.5
**Drank alcohol (of any type including surrogates) several** times or more in the year before death*		
Yes	142	84.0
No	18	10.7
Do not know	9	5.3
**Drank alcohol (of any type including surrogates) several** times or more in the month before death *		
Yes	84	53.8
No	45	28.8
Do not know	22	14.1
Missing answer	5	3.2
**Frequency of drinking any alcohol during last year**		
Every day/almost every day	48	28.4
1-4 times per week	47	27.8
3 times per month or less frequently	47	27.8
Never/almost never	18	10.7
Do not know	9	5.3
**Drank surrogates several times or more in the year** **before death**		
Yes	18	10.7
No	127	75.1
Do not know	21	12.4
Missing answer	3	1.8
**Smoking status at death**		
Smoker	132	78.1
Ex-smoker	15	8.9
Never smoked	22	13.0

**Total**	**169**	**100**

*This question was asked when deceased man was ever drunk alcohol in his life at least a few occasions (N = 156)

### Alcohol biomarkers and proxy reported drinking

Median values of the various alcohol biomarkers by proxy reported drinking behaviour are shown in Table 8. Median blood alcohol concentration was only raised substantially among daily drinkers, consistent with its rapid disappearance from the body. However, a more graded association was apparent for the novel biomarkers PEth in whole blood and EtS and EtG in urine, with negative or very low levels in reported abstainers. However, for a proportion of subjects where the proxy reported that the man had not drunk in the last month the biomarkers were clearly positive, suggesting proxy underreporting. The liver enzymes AST and ALT display a very wide range, consistent with a variable degree of post mortem haemolysis and autolysis of hepatocytes and, for AST, myocytes. In contrast, there was a significant association between frequency of drinking alcohol and GGT, although this was mainly due to elevated levels among daily drinkers.

**Table 8:** Biomarker levels (median and inter-quartile range (IQR) for proxy-reported alcohol drinking behaviour

Alcohol drinking behaviour of subject before death	Biomarker															
	
	Ethanol (blood) mg/g		Ethanol (urine) mg/g			AST (serum) U/L		ALT (serum) U/L		GGT (serum) U/L		PEth (blood) μmol/L		EtG (urine)mg/L		EtS (urine) mg/L
	
	N	Median (IQR)	N	Median (IQR)	N	Median (IQR)	N	Median (IQR)	N	Median (IQR)	N	Median (IQR)	N	Median (IQR)	N	Median (IQR)
**Frequency of drinking any alcohol during last year**																
Every day / almost every day	45	0.63 (0-2.0)	30	0.7 (0-2.9)	29	1558 (562-3210)	29	929 (237-2725)	29	193 (116-526)	44	9.7 (4.1-19.7)	26	78.3 (4.6-179)	26	23.1 (0-439)
1-4 times per week	46	0 (0-2.1)	40	0.6 (0-3.4)	30	972 (551-1573)	30	985 (355-2618)	30	69 (49-122)	38	8.7 (2.6-16.1)	33	43.4 (4.3-246.9)	33	23.0 (0.09-350)
3 times per month or more rarely	45	0 (0-2.1)	37	0 (0-2.2)	33	874 (467-2210)	33	910 (258-4675)	33	77 (62-111)	41	5.1 (0.5-19.0)	31	41.7 (0.2-338)	31	8.4 (0-198)
Never / almost never	16	0 (0-0)	10	0 (0-0)	11	1686 (895-2384)	11	1856 (1393-3319)	11	69 (30-172)	11	0 (0-0.4)	4	0.1 (0-0.2)	4	0.01 (0-0.15)
*P *value for trend*		0.041		0.080		0.897		0.237		< 0.001		< 0.001		0.046		0.038
**Drinking any alcohol during last month**																
Yes	80	0.2 (0-2.2)	58	0.6 (0-3.4)	54	1114 (634-3119)	54	988 (466-2940)	54	92 (57-168)	66	10.8 (4.3-22.9)	49	94.6 (4.6-338)	49	23.0 (1.1-69)
No	44	0 (0-2.0)	34	0 (0-2.2)	26	896 (399-1779)	26	885 (174-2725)	26	96 (66-172)	41	3.9 (0.2-10.3)	29	37.3 (0.3-172.2)	29	8.4 (0.1-49.8)
*P *value**		0.675		0.373		0.166		0.399		0.962		0.001		0.180		0.224
**Drinking surrogates during last year**																
Yes	16	0.3 (0-1.8)	11	0 (0-3.1)	11	1558 (283-6208)	11	1031 (237-1951)	11	255 (86-511)	16	9.7 (3.4-18.5)	10	138.5 (2.7-771)	10	33.3 (0.6-98.7)
No	12 1	0 (0-2.1)	95	0 (0-2.9)	83	1036 (553-2604)	83	1078 (359-3810)	83	85 (52-163)	103	6.7 (1.2-15.5)	74	51.9 (2.5-246.9)	74	12.6 (0.7-53.7)
*P *value**		0.912		0.553		0.922		0.202		0.010		0.362		0.349		0.355

*Regression method was applied for trend using log-values of biomarkers

**T-test was applied using log-values of biomarker

## Discussion and Conclusion

This study provides a unique resource for studying the existence of pathologies detected at forensic autopsy with particular emphasis upon the role of recent and chronic alcohol drinking in a country with a high level of hazardous drinking. These first results have shown that several of the novel alcohol biomarkers appear to perform well in post-mortem samples, and could be more widely used in other autopsy studies particularly in the absence of proxy-reported alcohol consumption patterns.

A major strength of this study is the systematic collection of information about the presence/absence of other pathologies of interest, regardless of whether or not they were considered to have contributed to the death. This goes considerably beyond what would be recorded routinely at forensic autopsy. A further strength is the systematic collection of proxy information about the deceased. However, the most unique feature of the study is the information on a wide range of informative alcohol biomarkers, several of which have not been used previously in this sort of post-mortem research study. The main weakness of the study is that it is restricted to deaths subject to forensic autopsy. These are a highly selected subset of all deaths, and thus cannot be used as a basis for estimating the overall burden of alcohol-related mortality. Furthermore, it is necessary to be aware of the potential for bias (such as recall and social acceptability) in the proxy interviews. Nevertheless, this sort of study can provide insights into the coexistence of multiple pathologies, and their relationship to patterns and levels of alcohol consumption.

One of the striking substantive findings is that over half of the deaths for whom proxy interviews were conducted were of men of non-Estonian (mainly Russian) ethnicity. This is a far higher proportion than seen in the national mortality data for men of working age, although it may partially reflect the fact that they are more likely to live in towns. However, we do know that non-Estonians have a higher mortality than Estonians for almost all causes of death in Estonia [24], and especially alcohol-related causes [25,26], consistent with known differences in binge [4] and surrogate drinking [7].

How do the results reported here compare to those from other autopsy studies? In a series of consecutive routine medico-legal autopsies in Norway 1973-1992 [27], 47.6% of autopsies had a BAC ≥ 0.5 mg/g. In our study, 55% of autopsies had a BAC ≥ 02 mg/g, which is the legal limit for driving a vehicle in Estonia [28].

This study substantially extends our understanding of the potential use of alcohol-related biomarkers at autopsy. We already know that liver enzymes, used widely to detect liver damage in living subjects, are of limited value post mortem. AST and ALT are increased by haemolysis and hypoxia and so are subject to differences in the process of dying. In contrast, GGT, as a marker of liver fibrosis and cirrhosis, is less affected by these processes.

Notably, PEth in blood, and EtG and EtS in urine all had zero or very low levels among the never/almost never drinking category. This indicates a high specificity of the novel alcohol biomarkers even in an autopsy setting. Nevertheless, the risk of post-mortem elevation needs to be carefully considered when interpreting values of PEth in post-mortem toxicology, given that some extremely high results were observed. Artefactual elevation of PEth levels may be due to ethanol produced by post-mortem fermentation in those who have not been drinking prior to death, and/or during storage in samples where the blood alcohol concentration at death was high [18].

EtG and EtS are conjugated ethanol metabolites. We have demonstrated [29,30] that EtG is sensitive to bacterial degradation (e.g. in the presence of E. coli) but also that EtG may be formed due to bacterial activity when ethanol is present. In contrast, EtS is not susceptible to bacterial degradation/synthesis making it the preferable biomarker for recent drinking in post-mortem toxicology. In routine clinical use, both metabolites are therefore measured as was done in this study. However, calculation of the EtG/EtS molar ratio, as an index of EtG disappearance/formation showed similar, albeit significantly higher values than in a Swedish clinical reference material (mean/median in the Estonian post-mortem sample were 2.57/2.39 mg/L; in the Karolinska reference sample 2.34/1.70 mg/L; *p *= 0.0064). There was only one sample that showed a markedly different ratio. However, this indicates that bacterial interference between time of death and autopsy was not a major problem in this study. Taken together, these data suggest that these novel biomarkers perform well as quantitative markers of the frequency of recent alcohol intake (i.e. within ~1-3 days) in aetiological epidemiological research. Despite this, their use in forensic legal contexts still requires caution [31].

In summary, this paper describes a unique resource for investigating the contribution of alcohol to premature mortality. The data are being further exploited to look at a number of key areas: 1) a more detailed exploration of the association between morphological pathologies, biomarkers of alcohol consumption and proxy reports of drinking behaviour; 2) assessment of the association of autopsy evidence of alcohol-induced damage and proxy reports and biomarker indicators of high levels of alcohol consumption; 3) investigating the association of proxy reports and biomarker indicators of alcohol consumption with mortality from cardiovascular conditions.

We hope that the approach that we have described will be applied by other research groups in other settings, thus helping to improve our still surprisingly limited knowledge of the post-mortem findings associated with problematic and hazardous alcohol use.

## Competing interests

The authors declare that they have no competing interests.

## Authors' contributions

The idea for the study was developed by MV, KL, MM and DAL. The forensic protocol was developed by MV and JT with input from all other authors. The interview study protocol was developed by KP and KL with input from MM and DAL. Data management and logistics were managed by IR, KL and KP. MT undertook the main laboratory work in Tartu. AH undertook the alcohol biomarker assays in Stockholm. Statistical analysis was done by IR. IR, JT and KP led the drafting of the manuscript. All authors contributed to revisions and interpretation of the results. All authors read and approved the final manuscript.

## Pre-publication history

The pre-publication history for this paper can be accessed here:

http://www.biomedcentral.com/1471-2458/12/146/prepub
